# The Canadian Childhood Nephrotic Syndrome (CHILDNEPH) Project: overview of design and methods

**DOI:** 10.1186/2054-3581-1-17

**Published:** 2014-07-22

**Authors:** Susan Samuel, Shannon Scott, Catherine Morgan, Allison Dart, Cherry Mammen, Rulan Parekh, Alberto Nettel-Aguirre, Allison Eddy, Rachel Flynn, Maury Pinsk, Andrew Wade, Steven Arora, Geneviève Benoit, Martin Bitzan, Robin Erickson, Janusz Feber, Guido Filler, Pavel Geier, Colette Girardin, Silviu Grisaru, James Tee, Kyle Kemp, Michael Zappitelli

**Affiliations:** University of Calgary, Alberta Children’s Hospital, 2888 Shaganappi Trail NW, Calgary, T3B 6A8 AB Canada; University of Alberta, Edmonton, AB Canada; University of Manitoba, Winnipeg, MB Canada; University of British Columbia, Vancouver, BC Canada; University of Toronto, Toronto, ON Canada; McMaster University, Hamilton, ON Canada; Centre Hospitalier Universitaire de Sainte-Justine, Université de Montréal, Montreal, QC Canada; McGill University, Montreal, QC Canada; University of Saskatchewan, Saskatoon, SK Canada; University of Ottawa, Ottawa, ON Canada; University of Western Ontario, London, ON Canada; Centre Hospitalier Universitaire de Sherbrooke, Sherbrooke, QC Canada; Dalhousie University, Halifax, NS Canada

**Keywords:** Nephrotic syndrome, Cohort study, Practice variation, Qualitative methods

## Abstract

**Background:**

Nephrotic syndrome is a commonly acquired kidney disease in children that causes significant morbidity due to recurrent episodes of heavy proteinuria. The management of childhood nephrotic syndrome is known to be highly variable among physicians and care centres.

**Objectives:**

The primary objective of the study is to determine centre-, physician-, and patient-level characteristics associated with steroid exposure and length of steroid treatment. We will also determine the association of dose and duration of steroid treatment and time to first relapse as a secondary aim. An embedded qualitative study utilizing focus groups with health care providers will enrich the quantitative results by providing an understanding of the attitudes, beliefs and local contextual factors driving variation in care.

**Design:**

Mixed-methods study; prospective observational cohort (quantitative component), with additional semi-structured focus groups of healthcare professionals (qualitative component).

**Setting:**

National study, comprised of all 13 Canadian pediatric nephrology clinics.

**Patients:**

400 patients under 18 years of age to be recruited over 2.5 years.

**Measurements:**

Steroid doses for all episodes (first presentation, first and subsequent relapses) tracked over course of the study. Physician and centre-level characteristics catalogued, with reasons for treatment preferences documented during focus groups.

**Methods:**

All patients tracked prospectively over the course of the study, with data comprising a prospective registry. One focus group at each site to enrich understanding of variation in care.

**Limitations:**

Contamination of treatment protocols between physicians may occur as a result of concurrent focus groups.

**Conclusions:**

Quantitative and qualitative results will be integrated at end of study and will collectively inform strategies for the development and implementation of standardized evidence-based protocols across centres.

## Abrégé

**Contexte:**

Le syndrome néphrotique est une néphropathie fréquente chez l’enfant, qui cause une morbidité considérable en raison de la récurrence d’épisodes de protéinurie importante. La gestion du syndrome néphrotique de l’enfant varie énormément d’un médecin et d’un centre de soins à l’autre.

**Objectifs:**

L’objectif principal de cette étude est de déterminer les caractéristiques associées à l’exposition et la durée du traitement aux stéroïdes, liées au centre, au médecin et au patient. En deuxième lieu, nous déterminerons la corrélation entre la dose et la durée du traitement aux stéroïdes, puis la durée avant la première rechute. À cette étude s’ajoutera une étude qualitative avec des groupes de discussion composés de professionnels de la santé qui viendra enrichir les résultats quantitatifs en favorisant une meilleure compréhension des attitudes, des croyances et des facteurs contextuels locaux qui entraînent des variations dans les soins.

**Type d’étude:**

Une étude méthodologique mixte; étude d’observation de cohorte prospective (composante quantitative), combinée avec des groupes de discussion semi-structurés composés de professionnels de la santé (composante qualitative).

**Contexte/Échantillon:**

Étude nationale, constituée des 13 cliniques canadiennes de néphrologie pédiatrique.

**Participants:**

400 patients âgés de moins de 18 ans, à recruter sur une période de 2,5 années.

**Mesures:**

Suivi des doses de stéroïdes pour chacun des épisodes (première présentation, première rechute et suivantes), tout au long de l’étude. Catalogage des caractéristiques liées au médecin ou au centre, et attestation des raisons justifiant les préférences de traitement au cours des séances avec les groupes de discussion.

**Méthodes:**

Tous les patients suivis de manière prospective dans le cadre de l’étude, dont les données constituent un registre prospectif. Un groupe de discussion à chaque endroit, afin d’enrichir la compréhension des variations dans les soins.

**Limites de l’étude:**

La contamination des protocoles de traitement entre les médecins peut se produire en raison de la tenue simultanée de groupes de discussions.

**Conclusions:**

Les résultats quantitatifs et qualitatifs seront intégrés à la fin de l’étude et permettront de mettre en place des stratégies de développement et de mise en œuvre de protocoles normalisés et fondés sur des données probantes.

## What was known before

Management of childhood nephrotic syndrome is variable between physicians and nephrology centres.

## What this study adds

This is the first national, Canadian, population-based evaluation of treatment of children with nephrotic syndrome. The study will provide novel information regarding factors driving treatment variation and how variation affects patient outcomes.

## Background

Childhood nephrotic syndrome, typically characterized by recurrent episodes of heavy proteinuria and oedema, [1] is one of the most common chronic conditions treated by paediatric nephrologists. Patients may experience significant morbidity from complications of the disease or its treatment including severe oedema, infections, and thromboembolism that often leads to frequent hospitalizations and utilization of health care services [2].

Steroids are the treatment of choice both for first presentation and for subsequent relapses of nephrotic syndrome [3–7]. Steroid treatment is supported by evidence published in systematic reviews [8, 9] and a recent international Clinical Practice Guideline [10]. Clinical response to steroids is the most important predictor of clinical outcome and prognosis [6].

Relapses of proteinuria are common in nephrotic syndrome and require multiple courses of steroid therapy. Relapse risk is tightly linked to cessation of steroids (i.e. when steroids are stopped, the likelihood of a relapse increases). Therefore, steroid treatment duration is a key determinant of patient outcomes including treatment and prevention of relapses and also steroid toxicity [11].

Although the current approach to treatment is based on several seminal studies, [4, 12, 13] patient management (choice of drugs, doses and duration) is known to be highly variable between physicians and care centres [14, 15]. A better understanding of the factors driving this variation will impact the design of future clinical trials evaluating optimal duration of steroid therapy to minimize relapses and toxicity.

Recently, treatment recommendations using the best available evidence for effective treatments for nephrotic syndrome were published in the Kidney Disease Improving Global Outcomes (KDIGO) Glomerulonephritis Clinical Practice Guideline [10]. In a physician survey of practice patterns, we found differences between reported practice and best evidence in the treatment of childhood nephrotic syndrome, and specifically in the duration of steroid therapy for first presentation of nephrotic syndrome [16]. Although, our survey was conducted prior to release of the Guideline, it is vital to understand what hinders and what facilitates the translation of Guideline recommendations into routine clinical practice, and these findings will also provide valuable insight into any research conducted in this area.

**Figure 1:**
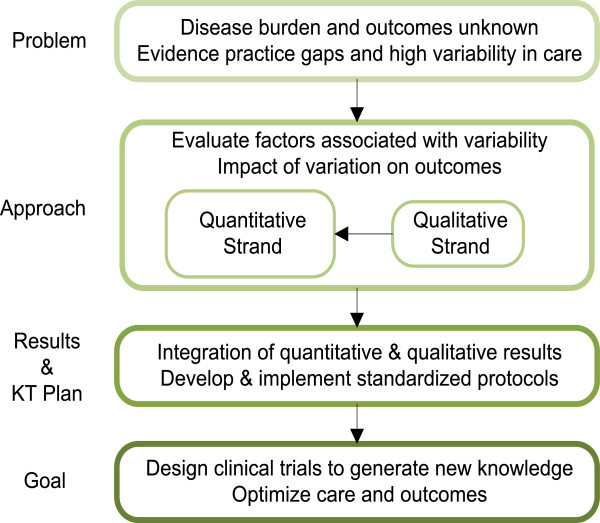
**Overview of the Canadian Childhood Nephrotic Syndrome Project.** *KT:* knowledge translation.

We hypothesize that physician and centre factors will play an important role in determining variation of steroid dosing for children with nephrotic syndrome. We propose to use a mixed methods design with both quantitative and qualitative components to study this problem [17]. The larger study is quantitative and has a longitudinal cohort design, while a qualitative study is embedded within the larger study to provide a deeper understanding of the complex and multi-level processes that lead to the variation in practice. In this article, we provide details regarding our study, the Canadian Childhood Nephrotic Syndrome (CHILDNEPH) Project, the team, and supporting infrastructure. See Figure 1 for an overview of the study.

## Methods

### Infrastructure and team

We applied the Knowledge to Action Cycle, an established framework for knowledge translation, to the management of childhood nephrotic syndrome [18, 19]. Using this framework, we engaged decision-makers and end-users (mainly paediatric nephrologists) in the research process by first setting priorities and identifying key research questions. These same individuals will also assist us in the interpretation and dissemination of results.

Our preparatory work was conducted within the Canadian Kidney Knowledge Translation and Generation Network (CANN-NET; http://www.cann-net.ca), a national initiative funded by the Canadian Institutes of Health Research (CIHR) linking kidney researchers, knowledge translation specialists, and knowledge users to ensure best practice in nephrology. CANN-NET has a Paediatrics Committee, with broad representation from Canadian paediatric nephrology centres, which is dedicated to promoting research, knowledge translation and best practices in paediatric nephrology. Following a survey of Canadian paediatric nephrologists in 2011, the Committee recognized the importance of knowledge generation and translation in childhood nephrotic syndrome. The CHILDNEPH Project was formed in response to this identified need and brings together researchers and knowledge users in paediatric nephrology utilizing national supporting infrastructure.

The Canadian Association of Paediatric Nephrologists (CAPN; http://www.capneph.ca) has supported our work by allowing us to engage the wider CAPN membership through study updates at semi-annual meetings. All 13 academic paediatric nephrology centres in Canada have agreed to participate (Table 1), with site investigators identified to ensure nationwide success of the study.

**Table 1:** **CHILDNEPH participating sites**

Participating Site (Hospital)	City
Alberta Children’s Hospital	Calgary
Montreal Children’s Hospital	Montreal
Stollery Children’s Hospital	Edmonton
Children’s Hospital	Winnipeg
BC Children’s Hospital	Vancouver
SickKids Hospital	Toronto
Royal University Hospital	Saskatoon
IWK Health Centre	Halifax
Centre Hospitalier Universitaire Sainte-Justine	Montreal
Centre Hospitalier Universitaire de Sherbrooke	Sherbrooke
McMaster Children’s Hospital	Hamilton
Children’s Hospital of Eastern Ontario	Ottawa
Children’s Hospital, London Health Sciences Centre	London

### Study design

**Figure 2:**
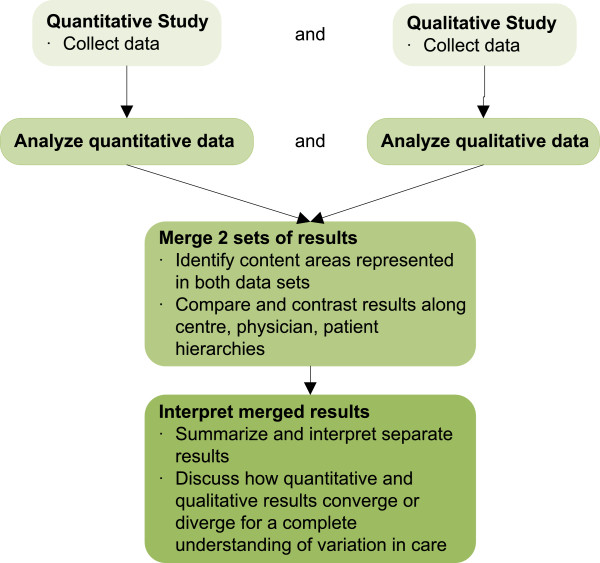
**Integration of qualitative and quantitative components of CHILDNEPH Project - Depiction of how the quantitative and qualitative components of the project will be complementary in answering research questions.**

We will use a mixed methods study as outlined by Creswell and Plano Clark [17]. Institutional ethics approval will be obtained for each participating site. We will conduct a national prospective longitudinal observational cohort study of children with nephrotic syndrome to obtain data about the different doses and durations of steroid treatments using a multi-level model analytic approach. We will also evaluate the effect of variation in steroid treatment on patient outcome (time to first relapse). A supplementary embedded qualitative study utilizing health care provider focus groups will enrich the quantitative results by providing an understanding of the attitudes, beliefs, and local contextual factors driving variation in care. The studies (3-year quantitative and 1-year qualitative) will be conducted concurrently. Upon study completion, we will use convergent parallel mixed methods analytic approach (both quantitative and qualitative data are collected and analysed during the same phase of research, with the two sets of results merged later for an overall interpretation) [17] to compare qualitative and quantitative data across centre-, physician-, and patient-level attributes (Figure 2).

### Quantitative study

#### Study period and subject selection

Site-specific patient identification protocols will be developed to ensure all patients are screened for eligibility. The enrolment period will last 2.5 years and the observation period will continue for an additional 6 months thereafter.

The developed inclusion criteria balance the need to maximize sample size, while minimizing individual contamination from prior treatments:

- A child upon first presentation of nephrotic syndrome who meets the following criteria: age: 1 to ≤18 years, oedema present, proteinuria (≥3+ on dipstick, ≥3 g/L on urinalysis or urine protein to creatinine ratio [U_P/C_] ≥200 mg/mmol), serum albumin ≤25 g/L, and no prior treatment with steroids for nephrotic syndrome.
- A child with an established diagnosis of idiopathic childhood nephrotic syndrome who presents at the beginning of either a first or second relapse (defined as proteinuria ≥3+ on dipstick, ≥3 g/L on urinalysis or U_P/C_ ≥200 mg/mmol for 3 consecutive days, after remission attained from prior treatment with steroids, during enrolment period prior to start of steroid-sparing agents).

Patients will be excluded if they meet one of the following criteria either at enrolment or during the course of study:

- Nephrotic syndrome in association with known disease entity (e.g. lupus, malignancy).
- Reduced serum C3 concentration.
- Steroid-resistant patients will be excluded from the final analysis (see below), but data will be entered into the registry for ongoing follow-up.

Defining an exclusion criterion after enrolment is necessary since current practice assumes that all patients are ‘steroid sensitive’ at first presentation. As mentioned above, a failure to induce complete remission of proteinuria defines steroid resistance. However, the protocol of this study will not define steroid resistance but rather allow clinicians to follow their local operational definitions.

#### Outcomes

Our primary outcome of interest is cumulative steroid exposure (in mg/m^2^ prednisone equivalents) per episode. An episode is defined as time from start of full dose daily steroid therapy (60 mg/m^2^ or 2 mg/kg up to a maximum dose of 60 mg/day) to either (i) cessation of steroids or (ii) re-start of full-dose steroids in steroid-dependent patients and is characterized either as first presentation or relapse. We will examine: a) total dose received per episode; and b) average daily dose per episode. This outcome definition is consistent with measures of steroid exposure used in prior studies conducted in Europe and Canada [7, 20]. Expressing steroid exposure per unit time allows for the comparison of patients with variable follow-up times (subjects recruited in the beginning versus at the end of a fixed study period). We are evaluating steroid prescription per episode as opposed to cumulative steroid exposure over a specified observation interval because our primary aim is to study determinants of variability in steroid prescription for episodes of proteinuria rather than patient outcomes (toxicity, frequency of relapses) related to cumulative exposure of steroids. The secondary outcome is the length of episode in days, with daily versus alternate day therapy taken into account. It is also important to note that we will be capturing ‘prescribed steroids’ or ‘steroids taken as reported by family’. We will not be monitoring adherence using pill counts, tracking of prescription refills or other methods due to feasibility constraints. Nevertheless, we will monitor adherence based on self- or parent-report as a percentage of total prescribed medication ‘as taken’ and this proportion will be used in adjusted analyses as appropriate.

The following outcomes will be collected for descriptive purposes: relapse rate, choice of steroid-sparing agent stratified by clinical indication (frequently relapsing/steroid-dependent/other), relapse rate prior to kidney biopsy, and reasons for kidney biopsy. The following outcomes will be used to measure complications of steroid treatment: anthropometric changes during observation period (age- and sex-specific standard-deviation scores (z-scores) for height, weight, and body mass index [BMI]), hypertension (≥95^th^ percentile) during the observation period and requirement for and duration of antihypertensive therapy, and external manifestations of steroid toxicity evident during semi-annual study visits (cushingoid facies, hypertrichosis, cataracts, striae, acne)–the definitions of which are similar to those observed in prior nephrotic syndrome dosing studies [4].

#### Main determinant and exposure variables

We will study centre-, physician-, and patient-level variables as potential determinants of steroid exposure both by cumulative dose and length of treatment. Centre-level variable include ‘use of standardized protocols [yes/no]’. Physician-level variables of interest include location of nephrology fellowship training (Canadian and non-Canadian) and years in practice, both shown to significantly impact variability in practice patterns [16]. The following patient-level variables will be used: age, gender, ethnicity, hematuria at presentation, estimated glomerular filtration rate (GFR) at presentation.

#### Study time points

Our study data points will follow the clinical course of each patient. Children will be treated with steroids at each episode. Study time points have been chosen to balance the need to collect the required exposure and outcome variables with the desire to minimize study visits. They will be collected using standardized case report forms and include: 1) study entry; 2) beginning of all subsequent relapses; 3) end of first episode and end of all subsequent relapses; 4) biannual study visits; 5) start of steroid-sparing agents; 6) kidney biopsy; and 7) study-end visit. Physician assessments will be performed at the following study points: study entry, biannual study visits, study end.

#### Data collection strategy

Data collected at study entry are a part of routine clinical care and include demographic information, height, weight, blood pressure, blood test results (C3, albumin, cholesterol if available), urine protein values by dipstick (or urine protein to creatinine ratio), and medical history. Details concerning the steroid prescription (including dose and duration of initial dose and taper) will be collected at entry and at all subsequent episodes. Data for study points at the beginning and end of all relapses, start of steroid-sparing agents, and kidney biopsy will be obtained using one or all of the following methods: chart review, review of patient log books of daily urine protein measurements and steroid dose given (part of clinical routine), and phone calls to family to confirm details of steroid prescription received.

#### Analysis

We will use mixed effects models with fixed effects for centre, physician, and patient characteristics and random effects to account for physicians clustering within centre, patients clustering within physician, and episodes clustering within patients to study the association between selected exposures and outcomes (total per episode, average dose per episode and length of steroid exposure). We will describe all additional outcomes of interest by centre- and patient-level variables. Association between cumulative steroid exposure per unit time during first presentation and time to first relapse will be determined using time-to-event analyses. We will account for potential collinearity of centre and physician level variables with steroid exposure in our multi-level models.

#### Sample size and power considerations

Based on an estimate of 150 new Canadian paediatric patients with nephrotic syndrome per year, a consent rate of 72%, [21] an average relapse rate of one per year, and an estimated drop out of 10% (due to loss to follow-up or steroid resistance), we estimate an enrolment of at least 394 patients over a period of 2.5 years. These 394 patients will yield between 394 and 650 observations (episode at entry plus one additional relapse for approximately 70% of all participants during the observation period). There is no simple analytic formula to calculate required sample size for mixed effects model analysis with three random effects (physicians within centre, patients within physicians and episodes within patients). Given the expected sample of 394 subjects and a total of 8 explanatory variables plus 3 random effects (estimated as parameters in the model), our analysis will comply with the guideline of 10 observations (at the unit of analysis level) per variable [22].

### Qualitative study

The embedded qualitative study will enrich the quantitative results by providing details regarding attitudes, beliefs, and local factors driving variation in care not captured by quantitative approaches. Factors and interplay between factors in clinical decision-making may involve physicians’ training and their familiarity with the evidence base, practice environment, and unique patient case scenarios. Theoretically grounded in the *Ottawa Model of Research Use*, [23] a knowledge translation model, we will conduct focus groups at participating sites with health professionals caring for these patients to probe for reasons for the variation of steroid protocols used. Eight focus groups (4–6 participants per group) will be conducted, each lasting 60 minutes. Focus group participants will be identified by local site investigator and will include physicians, nurses and pharmacists who are involved nephrotic syndrome treatment at that site. Focus group questions are based upon the six components of the Ottawa Model of Research Use (innovation, potential adopters, practice environment, interventions, adoption, and outcomes) and employ the same multi-level framework as the quantitative study (centre-, physician-, patient-level). We will group study sites that within geographic proximity (e.g. all Quebec sites). In centres where there are only one or two individuals managing nephrotic syndrome, we will do telephone interviews using a modified interview guide. This data will be key for knowledge translation and dissemination strategies at the end of study.

#### Overview of qualitative data analysis

Qualitative data collection and analysis will proceed concurrently. The inductive analysis will occur in three phases: coding, categorizing, and developing themes [24]. Data analysis will be managed using the ‘NVIVO’ software package (http://www.qsrinternational.com). To minimize potential bias, the initial coding and analysis of the qualitative data (reviewing focus group transcripts) will be performed by members of the qualitative team who are not participating in the initial quantitative analysis. All investigators will be involved in the final stages of analysis as de-briefers to provide credibility to the findings. All methodological decisions and insights will be documented in an audit trail [24, 25].

### Project and data management

The study will be supervised and managed by a coordinating centre in Calgary, Alberta. All de-identified patient and physician data will be entered into an online multi-centre database (REDCap™) which allows users to build databases securely for the purposes of research, particularly longitudinal cohort studies.

### Integration of quantitative and qualitative studies

A convergent mixed methods analytic approach will be used to compare the results obtained in both the quantitative and qualitative analysis. Using matrices of the hierarchies of centre-, physician- and patient-levels, the explanatory quantitative variables and themes arising from the qualitative study (e.g. existence of standardized protocols in each centre, age and training background of the physician, patient’s age and ethnicity) at each level will be compared for their congruence.

### Funding sources

This project is funded by the CIHR Institute for Nutrition Metabolism and Diabetes (start-up funds, grant number PNI-134070), and the University of Calgary Roy Vi Baay Chair for Kidney Research.

## Discussion

The CHILDNEPH Project will address two important unanswered questions in childhood nephrotic syndrome–(i) Who and what drives variability in care? and (ii) Does the variability in care influence outcomes? We will also establish a national longitudinal cohort with a web-based collection of patient data. The study data and results will be used to systematically evaluate clinical care and outcomes, to help standardize care across centers and to evaluate the effects of such efforts on patient outcomes. We will also demonstrate the feasibility of identifying patients across Canada to conduct well-designed RCTs for optimal steroid therapy and steroid-sparing agents for nephrotic syndrome, while determining what barriers may exist in enrolling such patients.

Since childhood nephrotic syndrome meets the CIHR definition of a ‘rare’ disease (affects one person out of 2,000 or fewer), [26] almost no single centre or region in Canada has a sufficient number of patients to produce generalizable knowledge regarding effective treatments. First arising in the paediatric oncology community in the 1950s and improving paediatric oncology outcomes from almost universally fatal to a current survival rate of 82%, [27] multi-centre collaborative clinical research networks can overcome this barrier and improve health outcomes of children with rare diseases. Another example of a collaborative network includes the ImproveCareNow network (https://improvecarenow.org) for paediatric inflammatory bowel disease (IBD). With 25% of American paediatric gastroenterologists and 34 sites, the group has seen remission rates for Crohn’s disease and colitis improve from 49% to 78% in just 5 years [28–30]. Each of these sub-specialty areas found significant improvements through participation in the network, adherence to common ‘model’ care guidelines, and examination of the influence of practice variation on patient outcomes. The CHILDNEPH study will be foundational for the establishment of such a network for childhood nephrotic syndrome and for our future work in this area.

Variations in clinical practice may exist as a result of uncertainty in evidence or a lack of implementation strategies for existing clinical practice guidelines, the latter of which can be hindered at various levels - patient, physician, and health system [19]. As part of the knowledge translation activities for the CHILDNEPH project, we will develop an active dissemination and implementation strategy to help promote the uptake of clinical practice guidelines for the management of childhood nephrotic syndrome and may include the development of clinical pathways. This combination of information regarding factors driving practice variation and knowledge translation strategies will increase the likelihood of uptake of guideline recommendations.

Data from this study will provide us with information to design a clinical trial to determine the impact of cumulative steroid exposure at first presentation on time to first relapse and frequency of relapses. The duration of steroid therapy for first presentation of nephrotic syndrome is still debated within the literature; [31, 32] thus, the KDIGO Guideline recommends a wide interval for steroid therapy (daily for 6 weeks followed by 2–5 months of alternate day treatment). Our study will help us understand why physicians choose one steroid duration over another, which will identify barriers to adopting common protocols for steroid therapy. Furthermore, data regarding use and response to steroids will assist us in designing a clinical trial to determine the best steroid-sparing agent in frequently relapsing and steroid-dependent patients.

One significant source of bias in our study is due to physician participation in the focus groups as we are concurrently conducting the longitudinal observational cohort study. We acknowledge that contamination of treatment protocols may occur between physicians in one centre and centers may take measures to standardize care after the focus groups. We will monitor for this trend in the data.

In summary, this national longitudinal cohort study will generate novel data regarding determinants of variation in care and the effects of variation on patient outcomes. The study will integrate clinical care and patient-oriented research programs, similar to other collaborative clinical care and research initiatives in childhood cancer and childhood inflammatory bowel disease [27, 29]. Our future directions will include design and conduct of RCTs to address critical knowledge gaps related to the treatment of nephrotic syndrome.

## Abbreviations

BMI: Body mass index
CANN-NET: Canadian Kidney Knowledge Translation and Generation Network
CAPN: Canadian Association of Paediatric Nephrologists
CIHR: Canadian Institutes of Health Research
CHILDNEPH Project: Canadian Childhood Nephrotic Syndrome Project
GFR: Glomerular filtration rate
KDIGO: Kidney Disease Improving Global Outcomes
U_P/C_: Urine protein to creatinine ratio
RCTsm: Randomized controlled clinical trials.
